# Ten Tips to Hurdle the Injuries and Illnesses During Major Athletics Championships: Practical Recommendations and Resources

**DOI:** 10.3389/fspor.2019.00012

**Published:** 2019-08-21

**Authors:** Pascal Edouard, Andy Richardson, Andrew Murray, Jennifer Duncan, Danny Glover, Marianna Kiss, Frédéric Depiesse, Pedro Branco

**Affiliations:** ^1^Inter-University Laboratory of Human Movement Science (LIBM EA 7424), University of Lyon, University Jean Monnet, Saint-Étienne, France; ^2^Sports Medicine Unit, Department of Clinical and Exercise Physiology, Faculty of Medicine, University Hospital of Saint-Etienne, Saint-Étienne, France; ^3^Medical Commission, French Athletics Federation (FFA), Paris, France; ^4^Division de Médecine Physique et Réadaptation, Swiss Olympic Medical Centre, Centre de Médecine du Sport, Centre Hospitalier Universitaire Vaudois, Lausanne, Switzerland; ^5^European Athletics Medical & Anti-doping Commission, European Athletics Association (EAA), Lausanne, Switzerland; ^6^Institute of Cellular Medicine, Newcastle University, Newcastle upon Tyne, United Kingdom; ^7^Knowledge Translation Team, Sport and Exercise, University of Edinburgh, Edinburgh, United Kingdom; ^8^Human Performance Science Research Group, University of Edinburgh, Edinburgh, United Kingdom; ^9^Health Education Yorkshire and the Humber, Leeds, United Kingdom; ^10^Hungarian Athletics Federation (Magyar Atlétikai Szovetség), Budapest, Hungary; ^11^National Institute for Sport Medicine, Budapest, Hungary; ^12^Department of Physical Medicine and Rehabilitation, University Hospital of Martinique, Le Lamentin, France; ^13^Health and Science Commission, International Association of Athletics Federations (IAAF), Monaco, Monaco

**Keywords:** health protection, sports injury prevention, illness prevention, track and field, top-level athletes, injury risk, illness risk

## Abstract

Participating or winning a medal in major track and field (athletics) competitions is the goal of every athlete. However, health problems can impair sports performance and affect this dream. Therefore, we present ten tips to help hurdle the challenges of illness/injury at major athletics championships: (1) Prepare for travel (medical checking, vaccine, time-zone, jet lag, culture, food habits…), (2) Respect athlete characteristics and discipline specificity (sex, endurance/explosive), (3) Educate athletes and their entourages regarding prevention, (4) Vigilance of painful symptoms and subclinical illness markers, (5) Avoid infection risk (washing hands, safe food and drink, avoid contact with sick people…), (6) Train appropriately and optimally (physical conditioning, technical training, load management, and psychological preparation), (7) Health status (history of previous injuries, well-being in the month before championships), (8) Lifestyle (good sleep, regular hydration and nutrition with safe water/food, regular fruits and vegetables, improve recovery strategies…), (9) Environmental considerations (heat, cold, air cleaning, changes or climatic conditions…), (10) Safety (equipment, rules, own-practice in athletics, and extra-sport activities). These ten tips “PREVATHLES” are based on our field experience in addition to existing epidemiological and experimental literature in athletics and other sports. Although there is currently no scientific evidence for their efficacy, sound judgement, and logical practice provide a strong basis, and given the low risk of using them in the benefit/risk balance, we suggest athletes and those around them follow these ten tips to limit the impact of injury/illness on championship performance.

## Introduction

Participating at major athletics events, such as Olympic Games, World or Continental Championships, is the goal for all track and field (athletics) athletes, aiming for optimal performance and winning medals. However, these dreams can be affected by health problems that impair sports performance.

During major athletics championships, a significant number of injuries and illnesses have been reported, varying according to the championship type (i.e., world/continental, outdoor/indoor, and/or adult/junior/youth championships), athlete sex and athletics discipline (Feddermann-Demont et al., 2014; Edouard et al., 2015b, 2019b; Edouard et al., under revision). In addition, athlete health status in the month before the championships has been reported to influence the occurrence of new injuries and/or illnesses during the championships period (Alonso et al., 2015; Edouard et al., 2015c; Timpka et al., 2017). These epidemiological results are fundamental to improve the development of more focused injury and illness prevention research and to implement preventative measures based on objective measures of the problem (van Mechelen et al., 1992). Epidemiological data provides useful insight when trying to anticipate medical service provision and to help in the screening of athletes at risk (Edouard et al., 2015a, 2019a,b; Edouard et al., under revision). Alongside surveillance, there is a need to develop prevention measures. Injury and illness prevention involves athletes as well as the team around them such as coaches, managers, family, sponsors, and includes health professionals and governing bodies (International Olympic Committee (IOC) (Engebretsen and Steffen, 2015), International Association of Athletics Federations (IAAF), European Athletics (EA), and national athletics federations) (Edouard et al., 2014, 2015a). People in a wide variety of roles can help decrease illness and injury in athletes.

Therefore, aiming to decrease illness and injury at major athletics championships, and especially at the 2019 IAAF World Championships, there is a need to propose prevention measures. To the best of our knowledge, currently there is no scientific evidence proven by randomized controlled trials or other high-quality studies on the efficacy of injury and illness prevention measures in athletics, especially in the context of major championships. Using an evidence based approach combining evidence from other sports and our experience in athletics, we present ten tips with materials/resources (Barton and Merolli, 2019) that might help to reduce the risk of injury and illness in championship preparation and competition.

## Prepare the Travel

Major championships often take place outside of the athlete's own country. Consequently, there is a need for travel, which may be short (i.e., a few hours and/or no time zone change) or long (i.e., more than 5 h and/or >4–5 time zone changes). Travel preparation includes different phases, with pre-travel, traveling, destination, and return (Mahadevan and Strehlow, 2017; Lohr et al., 2018).

Before traveling, athletes and their entourage (especially coaches and medical teams) need to be prepared for practical aspects, including the travel schedule, baggage, accommodation, sporting calendar, insurance, etc. They also should be prepared for the medical aspects, such as the vaccine requirements and health operations in that particular destination, in addition to awareness of nutritional habits (i.e., foods and water), environmental conditions (i.e., temperature, humidity, pollution, and altitude), endemic pathogens, sanitation standards, and cultures that they are likely to encounter (Mahadevan and Strehlow, 2017; Lohr et al., 2018). One practical application could be to anticipate the need for extra rooms to isolate unwell athletes.

During the traveling phase it is important to prevent the negative effects of jet lag, such as sleep disruption, fatigue, and dehydration, which can affect health (i.e., impaired immune function and increase illness risk) and sports performance (Manfredini et al., 1998; Schwellnus et al., 2012; Fowler, 2016; Walsh, 2018). In order to minimize jet lag and travel fatigue effects when traveling, especially >4–5 time zones, we suggest (Schwellnus et al., 2012; Fowler, 2016; Mahadevan and Strehlow, 2017; Schwellnus, 2019):

- arrive at the destination at least 1 day early for each time zone crossed,
- before departing, attempt to partially synchronize sleep/wake cycles and meals for a few days,
- during the travel, avoid sleep deprivation, exposure to dry cabin air, and avoid prolonged relatively immobilized positions, and use appropriately screen (i.e., blue light) and dark glasses to help synchronize sleep/wake cycles with that of the destination,
- on arrival prioritize exposure to sunlight and participate at social activities and training sessions as soon as possible according the usual schedule of the destination, and use regular sleeping and eating times appropriate to the arrival schedule.

The use of sleeping medications or melatonin should only be considered after medical prescription, and/or if medications were already used by the athlete. And remember to consider these suggestions also when traveling back home.

## Respect Athlete Characteristics and Discipline Specificity

Since Edouard et al. (2015b) reported sex-related differences in injury risk, we suggest the need to consider a sex-related approach in injury prevention measures. Male athletes suffered more thigh, lower leg, hip/groin injuries and muscle strain and muscle cramps, with female athletes experiencing more stress fractures (Edouard et al., 2015b). Thus, injury prevention measures should focus more on functional conditioning, biomechanical improvements, effective regeneration, and workload optimisation, and should target the lower extremity muscles for male athletes, whilst focusing on stress fracture prevention in female athletes.

Athletics is composed of various disciplines with different physical, mechanical, technical, and psychological demands (Edouard et al., 2011, 2015a,b; Feddermann-Demont et al., 2014), which result in different injury patterns (Edouard et al., under revision). During international athletics championships, the highest injury rates were reported in combined events for male and female athletes, and lowest in throws for male and female athletes, as well as race walking for female athletes (Edouard et al., under revision). Injury patterns significantly varied between disciplines for location, type, cause, and severity (Edouard et al., under revision). Thigh muscle injuries were the main injury diagnoses in sprints, hurdles, jumps, combined events, and race walking, in both male and female athletes (Edouard et al., under revision). Lower leg muscle injuries were the main injury diagnoses in marathon, and lower leg skin injury in middle and long distances, in both male and female athletes (Edouard et al., under revision). And trunk muscle and lower leg muscle injuries were the main injury diagnoses in throws in both male and female athletes (Edouard et al., under revision). Consequently, we suggest that (i) championships injury prevention preparation should be discipline-specific, and (ii) local organization and medical teams should take into account such information to anticipate medical service provision for the different disciplines during the competition (Edouard et al., 2018; Edouard et al., under revision).

Higher illness rates have been reported in endurance events compared to explosive disciplines during major outdoor athletics championships (Timpka et al., 2017; Edouard et al., 2019b). This difference was largely due to the higher rate of exercise-induced dehydration/fatigue/hypotension/collapse problems in endurance disciplines (Edouard et al., 2019b). We therefore suggest paying attention to endurance athletes, by taking into account the environmental/climatic conditions (e.g., heat, humidity, and wind) in order to prevent heat illnesses, as well as preventing respiratory tract and infection problems (Edouard et al., 2019a).

## Education

Athletes should be at the center of the illness and injury prevention. However, all stakeholders can positively impact and help implement prevention measures. Athletes, coaches and other members of the team need to be aware of the benefits of preventing injuries and illnesses, and to have an understanding of existing preventive measures. Preventative interventions are not likely to succeed without high levels of understanding and compliance. Medical teams, physiotherapists, psychologists, and dieticians should actively participate in the education of athletes and their entourage, as well as the governing bodies at international or national level, aiding the dissemination and promotion of preventative measures. Visual information is far more likely to be remembered than plain text. We advocate asking athletes in what format (for example oral presentation, infographics, and video) they would like to receive information. Communication between all stakeholders should be actively promoted.

## Vigilance of Pain Symptoms and Subclinical Illness Markers

Pain is a natural mechanism to protect against injuries (Tesarz et al., 2012), alerting us to actual, potential or imminent tissue damage (Moseley, 2016; Hainline et al., 2017). In sport, pain is a frequent complaint by the athletes, not always associated with injury/illness (Harringe et al., 2004; Bahr, 2009). Pain can be experienced as a physiological response to normal training (i.e., adaptation of tissues to exercises without any significant tissue damage), or as a physiological “warning signal” of a tissue damage (i.e., injury). Therefore, difficulties arise in the differentiation between “physiological” and “pathological” pain: “to distinguish the ‘warning signal' from the ‘noise' of pain” (Edouard, 2018). When athletes experience pain, they can choose to act protecting his/her body or choose to ignore the pain signal and continue with the sporting activities, hoping that the pain goes way.

We think that paying attention to the pain warning signal occurring during/as a consequence of sporting can help athletes prevent injuries. A functional diagnosis based on the level of pain and consequent level of impairment could be helpful for athletes, coaches, and health professionals to prevent tissue damages and injuries, without having a clear diagnosis. This enables load management strategies to attenuate or prevent increases in pain, with the aim of limiting tissue damage (Edouard, 2018). This is highly challenging in the context of elite/professional athletes given the need for high training loads. Nevertheless, we suggest being vigilant to pain, and not ignoring it.

Non-specific symptoms such as fatigue, myalgia or arthralgia, headache, and fever should be considered as warning signal of acute illness, but can also be symptomatic of over-reaching and overtraining (Schwellnus et al., 2016), and thus should not be ignored in a prevention strategy.

## Avoid Infection Risk

Thirty percentage of illnesses reported during international athletics championships were claimed to be caused by infections (Edouard et al., 2019b), and 46–76% during Summer Olympic Games (Engebretsen et al., 2013; Soligard et al., 2017). Consequently, promoting measures to reduce the spread of communicable infections is highly relevant in the context of international championships. This includes basic actions such as (Hanstad et al., 2011; Alonso et al., 2012; Schwellnus et al., 2016, 2017; Timpka et al., 2017; Edouard et al., 2019a,b):

- washing hands, especially before eating and after going to the toilet (https://www.cdc.gov/handwashing/),
- avoiding hand shaking,
- keeping people that are unwell at a distance, for example separating sick athletes from the healthy athletes by requesting that competition organizers book extra hotel rooms,
- consuming safe food and water (i.e., avoid undercooked meat, wash and peel fruit where needed, use bottled water for drinking and cleaning teeth where water is not deemed drinkable),
- promoting food rich in vitamins and minerals (e.g., fruits and vegetables),
- promoting good sleep,
- being up to date with standard and travel vaccinations (https://www.nhs.uk/conditions/vaccinations/),
- checking your health status with your doctor.

## Train Appropriately and Optimally

Optimal preparation for competition is fundamental to good performance and reducing the likelihood of injuries and illness (Schwellnus et al., 2016; Soligard et al., 2016). An appropriate training regime in athletics should include (Edouard et al., 2015a; Schwellnus et al., 2016; Soligard et al., 2016):

- physical and functional conditioning to improve sensorimotor control, by for instance stretching, muscular strengthening, particularly eccentric, proprioceptive, balance, increased resistance to fatigue,
- technical work to avoid technical mistakes that may result in injury,
- psychological work by for instance mental preparation, mental imagery, psychological input, etc. not forgetting optimal load management.

To our knowledge, no physical conditioning programmes have proven efficacy to decrease injuries in athletics, although examples exist in other sports (Lauersen et al., 2014). Some programmes have been proposed in athletics: eccentric exercises targeting the hamstrings (Malliaropoulos et al., 2012; Askling et al., 2014), “Decathlon of Injury Prevention” (by the Medical Commission of the French Athletics Federation: http://www.athle.fr/asp.net/main.html/html.aspx?htmlid=4175). The improvement of technical movements, as in the highly technical disciplines (e.g., pole vault and hurdles) seems of interest to prevent injuries (Rebella et al., 2008; Rebella, 2015). In this respect, efforts should be made to develop and validate injury prevention programmes (Edouard et al., 2015a).

## Health Status

When an athlete has a chronic disease (e.g., diabetes and epilepsy), it should be stabilized before the championships, alongside a management strategy for anticipated complications.

A history of previous injury has often associated with the occurrence of a new injury in sport (Hägglund et al., 2006). During major athletics championships, athletes who reported an injury complaint during the month before the championship had a 2- to 4-fold risk of sustaining a new injury during the period of the championship (Alonso et al., 2015; Timpka et al., 2017). Illness symptoms causing anxiety in the month before championships were associated with a 5-fold increase in the likelihood of in-championship injury (Timpka et al., 2017). As a basic practical application of such finding, we suggest that athletes, coaches, and medical teams should:

- improve rehabilitation procedure and general physical conditioning,
- continue regular conditioning after healing,
- be vigilant of recurring symptoms (i.e., attentive to pain, complaints, fatigue…),
- carefully follow athletes in final preparation of championships by for instance monitoring the athletes' health status before championships using a pre-participation health questionnaire (Alonso et al., 2015; Edouard et al., 2015c, 2018; Timpka et al., 2017).

Finally, to maintain good health status and include experience of previous health problems, we suggest optimal communication between athletes and the medical team, as well as other stakeholders around the athlete (e.g., coach, physical trainer, manager and club director, sports federation, agent/manager, family…), ensuring that medical confidentiality is maintained (Dijkstra et al., 2014; International Olympic Committee, 2016; Schwellnus et al., 2016). This can be summarized as “medical teams should know their athletes.”

## Lifestyle

A healthy lifestyle can help to prevent injuries and illnesses, including (Hirshkowitz et al., 2015; Irwin, 2015; Schwellnus et al., 2016; Walsh, 2018):

- good quality and quantity of sleep;
- regular hydration of safe water (i.e., closed bottle);
- appropriate nutrition with safe food, cooked meat, regular fruits and vegetables, wash fruit;
- improve recovery strategies;
- reducing life stress;
- avoid tobacco, excess alcohol, doping, and recreational drugs….

## Environmental

Temperature, humidity, pollution, or altitude can influence sport performance and health.

Major Athletics championships often take place during the summer, consequently athletes may have to exercise in the heat, exposing the athlete to an increased risk of heat illness (Alonso et al., 2012; Périard et al., 2017; Timpka et al., 2017; Edouard et al., 2019b). As prevention measure, in a guideline for athletes and their entourage to prepare the IAAF World Athletics Championships Doha 2019 and the 2020 Tokyo Olympic Games, Racinais et al. suggest preparing heat acclimatization, avoid dehydration, and adapt pre- and pre-cooling (Beat the Heat: https://www.iaaf.org/about-iaaf/documents/health-science).

In contrast, there is also evidence of cold extremes being linked to increased incidence of infection, with human rhinovirus thought to replicate more robustly in cooler nasal temperatures (Foxman et al., 2016).

Pollution could be a source of problems, especially in endurance disciplines where it has been linked to reduced sport performance and increase health problems (Rundell, 2012). To prevent illnesses due to pollution, prioritizing training far from vehicle pollution, and having a clear plan to manage poor air quality should it occur.

Major athletics events are rarely at high altitude, but athletes should prepare for the championships by training at the same altitude, adapting dietary strategies and with optimal psychological approach, alongside early arrival for acclimatization purposes (Burtscher et al., 2018).

## Safety

The last tip is to advise athletes to follow safe athletics practice, but also safe habits in their daily life (e.g., driving, food, or sexual habits) (Schwellnus et al., 2016, 2017).

For sport practice, we suggest using appropriate equipment, for example throwing cage and jumping mat should not be too small and should be in perfect condition in order to ensure security when athletes throw or jump. We suggest that the competition schedules should be appropriate to the circadian rhythm (e.g., not during the night) and to the weather conditions (e.g., not during, e.g., extreme heat, rain, storm….). To avoid accident with throws, we suggest avoiding the occurrence of two events at the same location (Edouard et al., 2015a). The medical services at competitions should be appropriately organized for the level of competition and can contribute to illness and injury prevention strategy (Pendergraph et al., 2005; Zemper, 2005; Edouard et al., under revision).

## Conclusions

**Figure 1:**
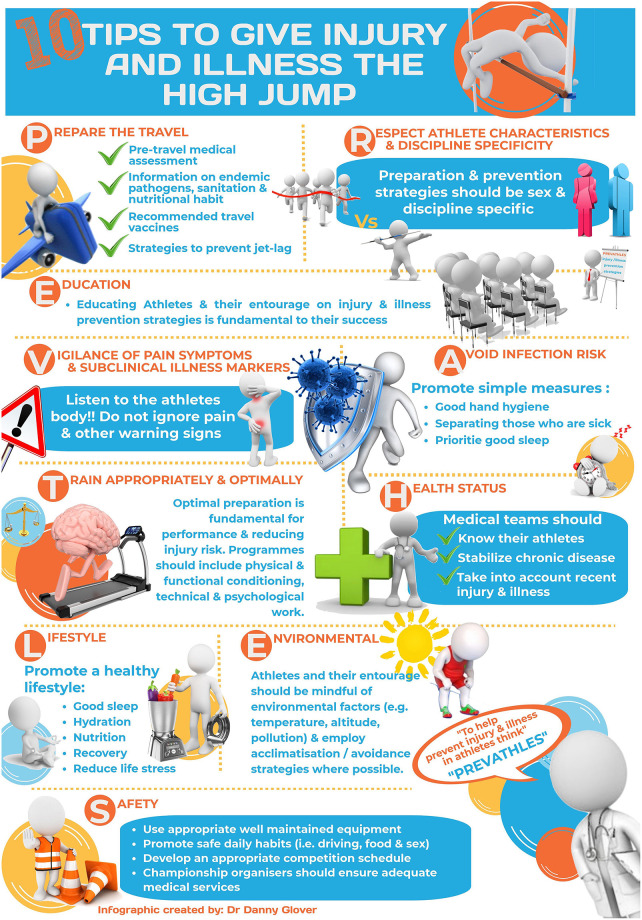
Infographic: ten tips to hurdle the injuries and illnesses during major athletics championships.

These ten tips are based on our field experience, and the evidence from epidemiological and/or experimental studies on athletics, together with extrapolations of studies in other sports. Their efficacy to prevent the occurrence of new injury and illness should be analyzed in order to promote their use. But before that, with sound judgement from basic and logical practice, and given the low risk in the benefit/risk balance, we suggest athletes and their entourage using these ten tips “PREVATHLES” (Table 1, Figure 1, and Supplementary Video 1) to try to limit the risk of injury and illness during championships. We hope these tips will help improve and optimize health protection and injury/illness prevention in athletes of all levels and have a subsequent benefit to overall competition performance. Efforts must be continued to progress in athletics injury and illness prevention!

**Table 1:** PREVATHLES: ten tips to hurdle the injuries and illnesses during major athletics championships.

**P**repare the travel
**R**espect athlete characteristics and discipline specificity
**E**ducation
**V**igilant of pain symptoms and subclinical illness markers
**A**void infection risk
**T**rain appropriately and optimally
**H**ealth status
**L**ifestyle
**E**nvironmental
**S**afety

## Author Contributions

PE conceived and designed the manuscript. PE, AR, and AM drafted the manuscript. DG identified the framework and produced the infographic. PE and AM commented on the infographic drafts and video drafts. JD identified the framework and produced the video. PE, AR, AM, JD, DG, MK, FD, and PB edited, critically revised the manuscript, and approved the final version.

### Conflict of Interest Statement

The authors declare that the research was conducted in the absence of any commercial or financial relationships that could be construed as a potential conflict of interest.

## Supplementary Material

Supplementary Video 1 PREVATHLES: ten tips to hurdle the injuries and illnesses during major athletics championships.
